# Insights into Nanopesticides for Ticks: The Superbugs of Livestock

**DOI:** 10.1155/2022/7411481

**Published:** 2022-06-08

**Authors:** Tean Zaheer, Muhammad Muddassir Ali, Rao Zahid Abbas, Khazeena Atta, Iqra Amjad, Anum Suleman, Zain Khalid, Amjad Islam Aqib

**Affiliations:** ^1^Department of Parasitology, University of Agriculture, Faisalabad 38000, Pakistan; ^2^Institute of Biochemistry and Biotechnology, University of Veterinary and Animal Sciences Lahore, 54000, Pakistan; ^3^Institute of Biochemistry, University of Punjab, Lahore, Pakistan; ^4^Department of Medicine, Cholistan University of Veterinary and Animal Sciences, Bahawalpur 63100, Pakistan

## Abstract

Livestock is an integral part of agriculture countries where ticks play significant role as potent pests causing considerable losses to economy and health. Drug resistance has made these pests supersede conventional therapies and control programs Nanotechnology here comes as an advancing and significant candidate alternatively able to reverse drug resistance. Nanoparticles, hence, against ticks may better be considered as nanopesticides that act in ways other than conventional drug efficacies. The methods of nanoparticles production include green synthesis, chemical synthesis, and arthropod-based synthesis. Pros and cons of these nanopesticides are by no means neglectable. Studies are fewer than needed to comprehensively discuss nanopesticides. Current review thus systematically covers aspects of ticks as livestock pests, their drug resistance, advent of nanotechnology against pests, their production methodologies, mechanisms of actions of ticks, and current limitations. This review opens several avenues for further research on nanoparticles as nanopesticides against ticks.

## 1. Introduction

Ticks are parasitic insects that feed on the blood of vertebrate animals. They are external, transient, and obligate parasites [1]. Hot and humid environments assist their survival, whereas cold temperatures hinder their development [2]. Ticks are classified into two families: Ixodidae and Argasidae. Due to the presence of a strong chitinous shield that completely covers upper surface of the mature male, they are called as hard ticks. The Ixodidae family is the most significant in causing diseases of livestock. The other tick family is the Argasidae. They are called soft tick since they do not have a shield [1]. Tick-borne viruses have evolved within the wild animals that act as hosts and reservoirs. In most cases, domestic animals come into contact with these reservoirs, either by the entrance of noninfested livestock into infested areas or the migration of diseased animals into noninfested areas, resulting in unstable situations [3]. Because of their capability to spread diseases to humans and animals, ticks had long been studied for their medicinal and commercially viable treatment options. Ticks cause enormous economic losses to cattle and have several detrimental impacts on the infected animals. Blood loss is caused directly by ticks acting as probable vectors for haemoprotozoa and helminth parasites. Ticks sucking blood in large numbers cause anemia and reduce weight gain in domestic animals, and their bites impair the quality of skin/hides. Ticks cause enormous losses to cattle due to their ability to spread protozoan, rickettsial, and virus-related illnesses, which are all economically significant globally. Ticks carry a number of pathogenic diseases that are hazardous to both humans and animals. Tick bites are mostly avoided with the usage of repellents and acaricides.

The evolution of resistance in ticks, toxic waste of the environment, and impurity in animal's milk and meat, on the other hand, are all serious issues related to pesticides. Alternative approaches become necessary in such situations inclusive of which are plants extracts and nanoparticles. Nanoparticles may be produced from plants and arthropods and by chemical methods. Among such metallic, metal oxide, and carbon nanoparticles are extremely effective to counter a range of arthropod pests. Toxicity studies of these nanoparticles have revealed wider range of response regarding sources of nanoparticles and their methods of production [4]. Challenges to use of nanoparticles like degradation, stability, and standardization make its commercialization difficult. Metallic nanoparticles, such as silver, titanium dioxide, zinc oxide, nickel, and copper, produced by green synthesis are considered less expensive and less harmful than those produced by chemical methods. Nanoformulations also protect the active ingredients from environmental deterioration and allow for fast tick penetration. Using nanomaterials to build rational and controlled pesticide release technologies could also increase pesticide loading, active ingredient dispersibility and stability, and their precise target ability [5]. Thus, it is high time to revisit and summarize nanopesticides, to estimate their safety and efficacy against one of the biggest livestock pests—the ticks.

## 2. Ticks as Livestock Pest

Ticks are parasites that can cause severe allergic reactions and carry a variety of viruses, bacteria, rickettsia, helminth and protozoa that can affect humans, pets, and livestock. Ticks are obligate blood-sucking ectoparasites that inflict skin irritation and injury as well as transmit infections to humans and animals such as tick-borne encephalitis, Powassan, Borreliosis, Lyme, Tularemia, Ehrlichiosis, Anaplasmosis, Babesiosis, and Theilerioses [6]. Being second to mosquitoes, ticks disseminate broader variety of pathogens than almost any classification of blood feeding insects on a global scale, impacting livestock, wildlife, pets, and humans [7]. Several tick species use humans as unintentional hosts. Tick bite protection largely relies on the utilization of synthetic insecticides and biopesticides [8]. Tick-borne diseases (TBDs) impact 80 percent of the world's largest cattle population, posing a serious threat to worldwide livestock production. There is a wider range of ticks infesting various hosts, while developing countries are suffering from this issue at higher rates because of a long list of associated risk factors. Many examinations on the threats of tick-borne disease have basically focused on a few variables, for example, natural (precipitation, temperature, and humidity), organic, and human elements (land use and creature cultivation). It has been reported that certain conditions of precipitation affecting humidity and temperature might impact tick load in animals. For instance, moderate rain and high humidity give conducive micro climatic conditions to mass proliferation of ticks and resulting invasion in animals [9].

The livestock sector is vital to rural communities that build their status and earn livelihood from rearing healthy animals. Tick control is usually done with chemical acaricides, which are expensive and potentially hazardous for environment and may lead to residues in animal products. Owing to the nonjudicious use of these acaricides, ticks are becoming resistant towards many acaricides. As a result, in the fight against ticks and tick-borne diseases, the application of ecologically friendly pesticides will be crucial. In certain areas of the world, ticks are one of the most perilous creatures followed by other ectoparasites causing morbidity and mortality in domestic animals and wildlife, capable of infecting associated human populations. Many tick-borne sicknesses are zoonotic. *Babesia bovis* and *Babesia bigemina* are apicomplexan protozoa that are spread by the invasive bovine ticks *Rhipicephalus (Boophilus) microplus* and *Rhipicephalus (Boophilus) annulatus*. Bovine babesiosis is harmful to cow health and costs the livestock business a lot of money in places such as Mexico where cattle fever ticks are still endemic [10]. A few ticks are obtrusive and shift microbes causing transboundary infections of high ramification for populaces of domestic animals. There is a need to develop more stringent strategies that are safer, cost-efficient, and sustainable for tick control. These strategies may further be extended in application from smaller scales to bigger scenarios [11].

## 3. Economic Burden and Resistance Pattern

An overwhelming burden of bacterial pathogens of livestock and pet animals [12–14] is being followed by ticks infestation. The tick *Rhipicephalus (Boophilus) microplus* among others is a large source of bacterial and protozoan infections causing direct and indirect economic losses within bovine populations worldwide. Tick-borne diseases have a considerable economic impact, which is growing every year. In 2002, the reported cost per patient diagnosed with Lyme disease in the United States was USD $8172 [6], which has increased to USD $11 838 in 2019 (CPI inflation calculator, https://data.bls.gov/cgi-bin/cpicalc.pl). Based on 42 743 cases reported to the Centers for Disease Control and Prevention (CDC) in 2017, a conservative estimate of the annual cost would be more than $500 million. Patients with posttreatment Lyme disease syndrome may face significantly higher expenses. Tick-borne diseases impact 80% of the world's cattle herd, costing between $13.9 billion and $18.7 billion annually. The economic losses owing to tick-borne disease were assessed at USD $364 million in Tanzania, with an estimated death of 1.3 million cattle due to theileriosis (68 percent), anaplasmosis (13 percent), and babesiosis (13 percent) [15].

Tick management has faced a variety of obstacles in recent decades, including the rapid development of resistance and the nontarget impact of chemical pesticides on public health and the environment. A range of control strategies are utilized to deal with these ectoparasites. Ecological, biological, and genetic techniques are among them. Arsenical, organochlorine, organophosphates, amidines, macrocyclic lactones, growth regulators, and phenyl pyrazoles are among common synthetic acaricides. Pyrethroids are extensively utilized against ticks. Pesticides used to control pests other than ticks on cattle can affect tick resistance and disease transmission. Resistance to various acaricide classes has been demonstrated in many countries where the ticks are prevalent. Tick control will assist farmers to save money and prevent losses caused by diseases spread by ticks. It is critical to suggest and develop innovative approaches to combat livestock ticks [16].

Ticks possessing ability to endure toxic drug dosages that are lethal for most ticks in a normal population of the same species are known as resistant. The interaction of acaricides with biological targets present in the tick causes their toxicity. Before the acaricide can work, it must go through a series of phases. It must encounter the arthropod, enter the body, be transformed into the active metabolite in some situations, and then be carried to the action site. As a result, any mechanism that alters one of these processes can result in resistance [17]. Medicinal plants offer abundant supply of the most environmentally beneficial insecticides for treating and managing animal parasites. Despite efforts to find dependable plant-based acaricides for ectoparasite control, efficient control of livestock ticks remains a key difficulty in modern veterinary entomology [18]. Several promising attempts to manage ticks of livestock and public health significance using green-fabricated nanoparticles have recently been made [19]. Nanoparticles may offer a new path for developing successful acaricidal formulations to combat ticks of animals.

## 4. Need for Nanotechnology

**Figure 1:**
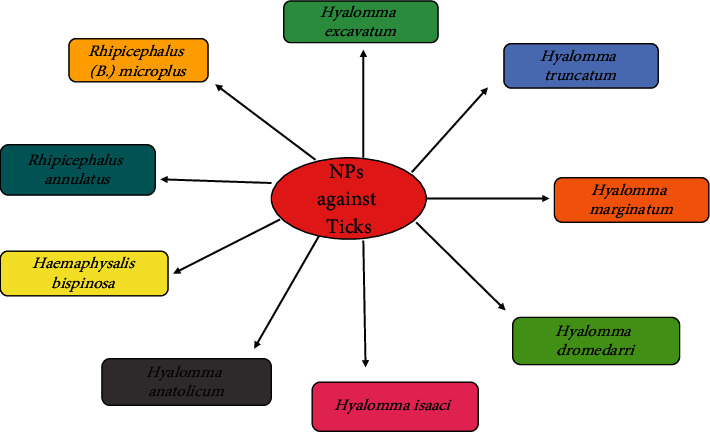
Overview of applications of nanoparticles (NPs) against ticks. In this review, we will explore nanoparticles as nanopesticides against ticks, but due to limited studies on ticks, prospective aspects will also be included.

The application of reliable control options against arthropod pests and vectors on a wider scale is a major problem for modern parasitology research [20, 21]. A potential convergence of nanotechnology and arthropod research has now unlocked new horizons for sustainable vector and pest management approaches. Nanoparticles have been considered as new insecticides that are poisonous to insect vectors and parasites that are important to global health (Figure 1) [22, 23]. When relative to chemical or physical techniques, periplasmic production of metallic nanoparticles facilitated by microbe-borne molecules and plants is a less expensive, one-step procedure that does not require the utilize of increased power, temperature, pressure, or deadly chemical compounds [24, 25]. During the production of metal and oxides of metal nanoparticles in green synthesis processes, metabolites from plant extracts or microbe filtrates potentially work as both reducing and stabilizing agents [23]. Metal, oxides of metal, and carbon nanoparticles were proven to be extremely potent often against economically important insect pest and carriers when generated using environmentally friendly methods [26].

More than 200 studies on the toxicity of nanoparticles to a variety of commercially important insect species have been published in the previous three years. And over half of them depended on nanoparticles made using the “green synthesis” method. Extracts from plants, fungus, bacteria, and their purified compounds, as well as dead insects, have been used to successfully decrease and maintain nanoparticles in aqueous phase. When compared to traditional physical and chemical synthesis processes, the green fabrication process has various advantages, including the absence of toxic substances and large energy inputs [27]. The total procedure is inexpensive, simple, and it is possible to obtain a wide range of nanomaterials, including gold, silver, titanium, zinc oxide, iron, palladium, and carbon nanoparticles. The nanoparticles have been successfully tested and designed against a variety of hazardous arthropods, including agricultural pests and vectors with public and livestock health implications.

The tremendous advances in nanopesticides research have prompted several international bodies to investigate potential difficulties related to the field use of nanotechnology. This investigation of current research tendencies might help identify research gaps and future goals. Polymer-based formulations have attracted the most attention in the previous two years, followed by formulations containing inorganic nanoparticles (e.g., silica and titanium dioxide) and nanoemulsions. The scarcity of data on nanopesticides efficacy is being researched, and several products have been demonstrated to be more effective than their commercial counterparts. The exact mechanisms of nanoparticle action are mainly unclear, and more research is needed before any conclusions could be drawn. There would be a greater incentive to develop nanopesticides that are less hazardous to the environment than traditional formulations, and further studies will be needed to determine any feasible products that could compete with present compositions in terms of cost and being effective. In the coming years, a significant amount of research will be required: (1) the establishment of accurate attributes through use of experimental techniques. (2) bioavailability and longevity of nanopesticides are being investigated, and (3) present climate threat assessment methodologies are being evaluated when necessary [28].

## 5. Approach of Nanopesticides

**Figure 2:**
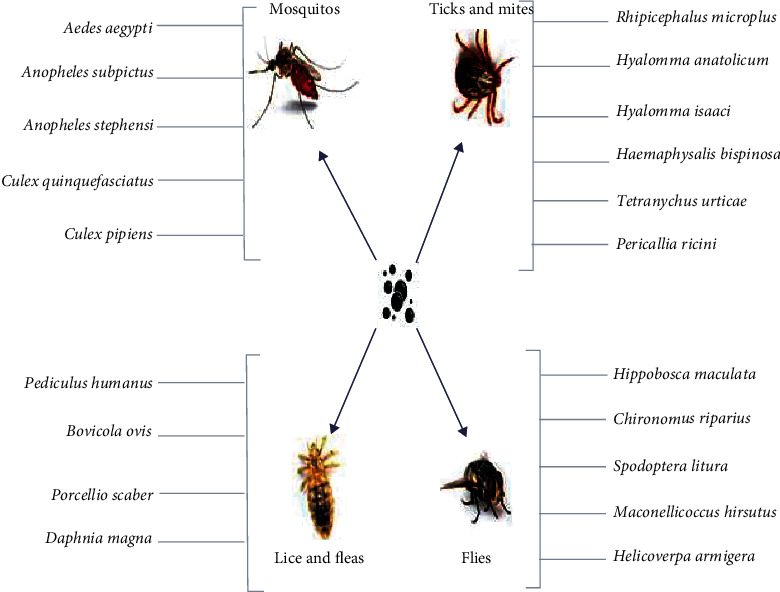
A summary of nanopesticides used against livestock pests.

Nanopesticides constitute either very small particles of a pesticide adjuvant or other small, designed structures with useful pesticide characteristics. A nanopesticide is a product that employs nanoscale work (e.g., the use of materials with at least one size dimension in the range of 1–100 nm) to improve efficacy or performance. These products, which are in various stages of research, can be used to boost the performance of current pesticide active ingredients, optimize their pollution control profiles, or do both. Nanopesticides have already been used against various livestock pests (Figure 2). Nanopesticides are relatively new technological production that carries several advantages in terms of pesticide use, including high working ability for lasting time and lower inclusion levels of hazardous active ingredients. Emulsions (e.g., nanoemulsions), nanocapsules (e.g., with polymers), and products containing pristine-engineered nanoparticles, such as metals, metal oxides, and nanoclays, have all been proposed as formulation types. The focus of testing these combination nanopesticides is on determining whether the presence of the nanoformulation brings potential changes in comparison to the active components of routinely used acaricides. The evaluation tests for estimation of effects of nanoparticles on off target organisms can help streamline the future of nanopesticides research avenues. Biochemical factor (BCF) is often used to evaluate possible dangers to high taxonomic groups and to classify substances as according to their durability, high adsorption potential, or cytotoxicity (PBT). BCF can be estimated as the ratio of the absorption order rate (Kin) to the different degradation rate constant. BCF can also be measured as the proportion of body residue to the stable concentration in water. The batch equilibration technique, in which the water and soil phases are blended to create phase equilibrium, is often used for measurements. The half-life (t1/2) or half-dissipation time (DT50) of a pesticide in soil can be evaluated in a laboratory incubation experiment or in an outdoor dissipation study [28].

### 5.1. Mode of Action of Nanopesticides

**Figure 3:**
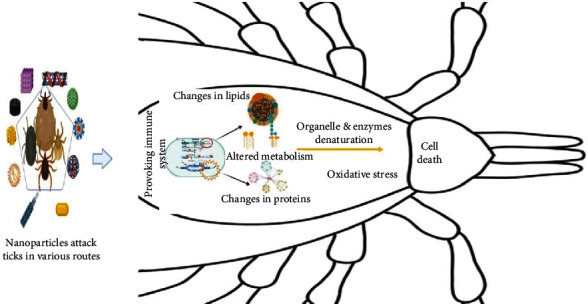
General mechanism of nanoparticles (nanopesticides) against ticks. Internally, nanoparticles activate the immune system of the pest causing changes in lipid, metabolism, protein, and cytotoxicity that inhibits growth and reproduction.

**Figure 4:**
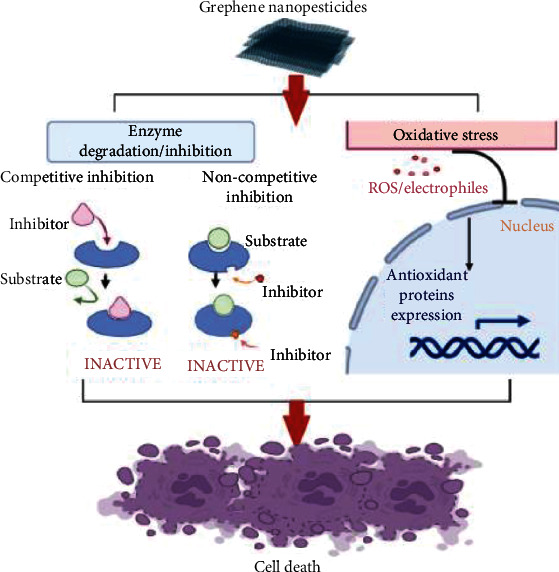
Mechanism of graphene nanopesticides against ticks. Graphene causes oxidative stress and cell death by the effect of antioxidant and detoxification enzymes.

**Figure 5:**
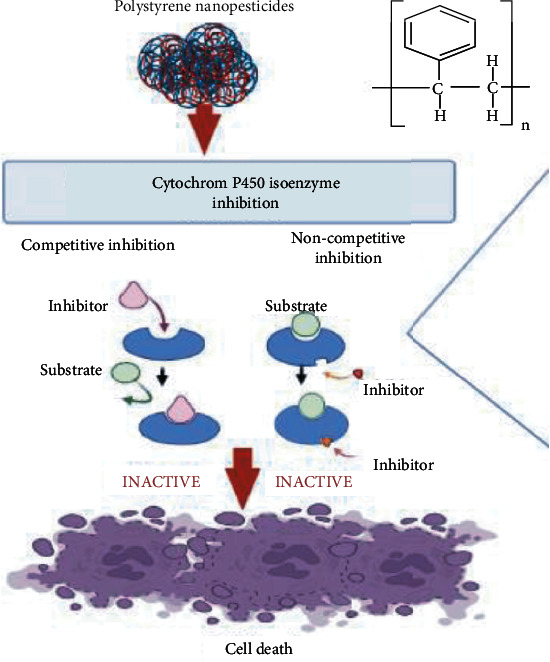
Mode of action of polystyrene nanopesticides against ticks. Polystyrene inhibits cytochrome P450 isoenzyme by competitive and noncompetitive pathway of enzyme inhibition. Enzyme inhibition leads to series of other reactions salient of which is oxidative stress that leads to cellular death.

**Figure 6:**
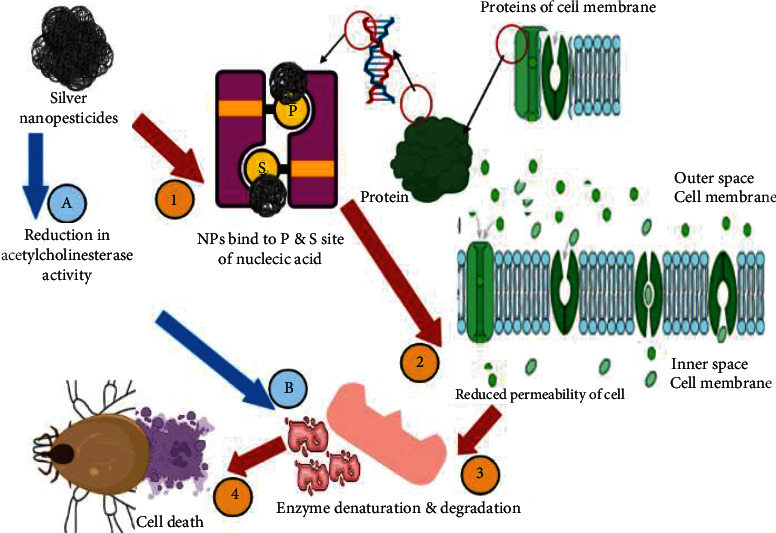
Mode of action of silver nanopesticides against ticks. Silver nanopesticides work by binding P and S region of nucleic acids and proteins (1) that leads to reduced permeability of cell membrane (2). This is further extended to enzyme degradation (3) which lastly takes cells to death (4). On other hands, silver nanoparticles also reduce acetylcholinesterase activity (A) that in turn also give rise to enzyme denaturation (B) that takes cell to death (4).

**Figure 7:**
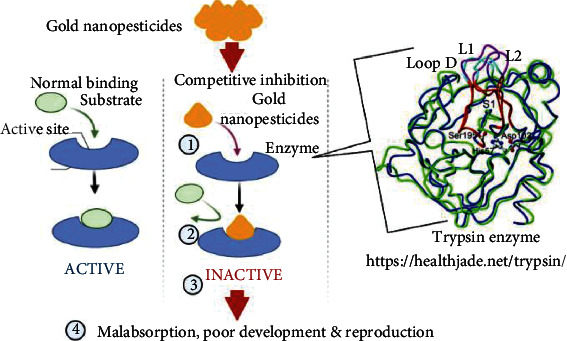
Gold nanopesticides' mode of action against ticks. Gold nanopesticides bind competitively with trypsin enzyme (1) and replaces substrate (2) leading to enzyme inactivation (3) that results in malabsorption, poor development, and reproduction of ticks (4).

**Figure 8:**
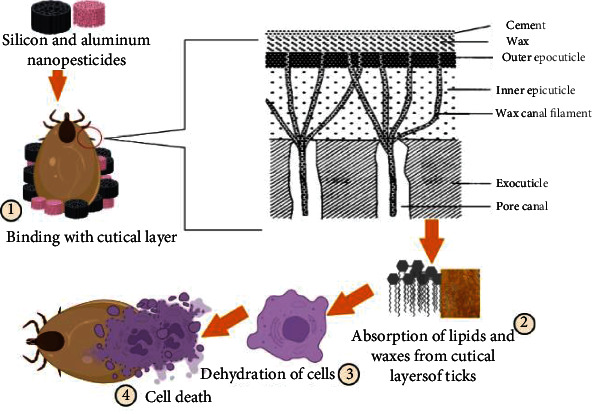
Silicon and aluminum nanopesticides against ticks. Silicon and aluminum nanopesticides bind with cuticle layer of ticks/insects (1) that results in physical absorption of lipids and waxes (2). This is ended with dehydration of cells (3) that finally results in cell death (4).

The utilization of nanomaterials made using a variety of synthesis procedures as new pesticides has recently piqued the scientific community's interest. Many studies have been carried out to test their toxicity against a variety of arthropod pests and vectors, with a focus on mosquitoes and ticks. Specific understanding on the mechanisms of action of nanoparticles against other insects and mites is limited. Nanopesticides act on the tick through various way, e.g., altering the lipids or proteins, creating oxidative stress, or disturbing the metabolisms of ticks (Figure 3). Silver and graphene oxide nanoparticles impact cancer prevention agents and detoxifying chemicals, causing targeted oxidative stress and cell death (Figure 4). CYP450 isoenzymes were suppressed by polystyrene nanoparticles (Figure 5), while acetylcholinesterase activity was reduced by Ag nanoparticles (Figure 6), and Au nanoparticles can affect the development and reproduction by acting as trypsin inhibitors (Figure 7) [20]. Metal nanoparticles may bind to the amino acids S and P in proteins and nucleic acids, decreasing the membrane permeability of cells and setting off organelle and thermal degradation, which may lead to cell death [29]. The cytotoxicity and genotoxicity cycles of silver nanoparticles have been more thoroughly investigated, probably owing to their surface property-dependent toxicity in organic models. Silicon and aluminum nanopesticides cause the cell death via absorption through cuticle layer and make the cells dehydrated thus leading to the cell death of ticks (Figure 8).

Active ingredient (AI) nanocapsules have exhibited controlled release and gradual breakdown qualities, making them more effective in managing pest. Various polysaccharide materials, such as chitosan, polyethylene glycol (PEG), starch, cellulose, and polyester substance, have been used in the synthesis of nanocapsules [30]. Nanomaterials including polymers, nanocrystals [31], lipid nanoparticles, and nanoporous silicas [32] have recently been used to generate nanopesticide formulations with higher potency and prolonged delivery. Nanostructured silicas, for example, have unique qualities such as high payload porosity, a sustainable framework, simplicity of manufacturing, and excellent biocompatibility [33] making them one of ideal for pesticide applications. Recently, nanopesticide formulations containing mesoporous silica monolith and mesoporous silica nanoparticles as carriers were discovered, with remarkable long-term release and distribution properties [34]. The silica shells may provide UV protection and serve as a good defense against photoinduced pesticide instability, according to a recent study that employed mesoporous silica hollow spheres to contain the biopesticide ivermectin. Photoprotection of pesticides such as deltamethrin using nanocarriers has also been documented [35].

## 6. Methods of Production

There are two majorly prevailing methods of preparation of nanoparticles. i.e., chemical method and green synthesis. However, other biological sources for instance microorganisms may also be used for this purpose. Arthropods may be used for preparation of nanoparticles and might be better option against pests of crops and livestock. A brief account of these methods is given.

### 6.1. Chemical- and Green-Synthesized Nanoparticles

Green synthesis of nanoparticles involves the use of bio agents such as plants and microorganism. Metal ions are reduced into NPs by plant metabolites such as terpenoids, tannins, alkaloids, steroids, saponins, polyphenols, alkaloids, phenolic acids, and proteins. Chemical-based nanoparticles are synthesized by reducing agents (organic and inorganic solvents). For the reduction of metal ions in aqueous or nonaqueous systems, several reducing agents such as sodium citrate, ascorbate, sodium borohydride (NaBH_4_), and elemental hydrogen may be used. The analysis of comparison between the methods is given in Table 1.

#### 6.1.1. Pros and Cons of Both Method of Nanoparticle Synthesis

By altering chemical quantities and reaction conditions, the morphological properties of nanoparticles (such as size and shape) can be modified (e.g., temperature and pH). However, when these synthesized nanomaterials are put to actual/specific applications, they may face the following limitations or challenges: (i) stability in hostile environments, (ii) lack of information about interactions with fundamental mechanism and modelling factors, (iii) bioaccumulation/toxicity characteristics, (iv) extensive analysis requirements, (v) need for experienced workers, (vi) problem in device gathering and structures, (vii) sustainable production (viii) reclaiming of substances and (ix) uniformity of reaction products [37]. Chemically produced nanoparticles require exceedingly expensive techniques of production. Furthermore, the ingredients utilized to make NPs, such as citrate, borohydride, thioglycerol, and 2-mercaptoethanol, are poisonous and dangerous [38]. Apart from these drawbacks, the produced particles are not of the expected purity, with chemical sedimentation on their surfaces. Preparing NPs with a well-defined size is difficult, necessitating a second procedure to prevent particle aggregation [39]. Furthermore, too many poisonous and dangerous by-products are produced during the synthesis. Cryochemical synthesis, laser ablation, lithography, laser irradiation, sono-decomposition, electrochemical reduction, thermal decomposition, and chemical reduction are all examples of chemical methods. Chemical synthesis of nanoparticles has the advantage of ease of manufacture, low cost, and high yield; nevertheless, chemical reducing agents are toxic to living organisms [40]. Green synthesis of nanomaterials, which is achieved through a process of regulation, control, cleanup, and remediation, may directly contribute to the environmental friendliness. Several elements, such as waste prevention/minimization, derivatives/pollution reduction, and the use of safer (or nontoxic) solvents/auxiliaries, as well as renewable feedstock, can thus be explained by some basic concepts of “green synthesis.” Green synthesis of nanomaterials may prevent the generation of undesirable or dangerous by-products by developing dependable, long-term, and environment friendly synthesis techniques. To attain this, suitable solvent systems and natural resources (such as organic systems) are required. Plant extracts are a relatively straightforward and easy procedure to generate nanoparticles at large scale when compared to bacteria and/or fungal mediated synthesis among the current green ways of synthesis for metal/metal oxide nanoparticles. Numerous physical and chemical production methods necessitate high levels of radiation, highly poisonous reductants, and stabilizing agents, all of which can harm nontarget species of living beings including humans and marine life alike. Green synthesis of metallic nanoparticles, on the other hand, is a one-pot or single-step ecofriendly bioreduction approach that starts with relatively little energy. This is also a cost-effective method of reduction [41–45]. These substances are naturally present in the living organisms used in the green synthesis approach for producing nanoparticles with biocompatibility. Bacteria are evident targets in the manufacture of nanoparticles due to their rapid development, low culturing costs, and ease of control and manipulation of the growing environment. At the same time, it is recognized that several bacterial species have unique mechanisms for reducing metal or heavy metal toxicity. Bacteria can synthesize nanoparticles both *in situ* and *ex situ*, which is why they are sometimes preferred. Metal ions can be reduced and precipitated for nanoparticle manufacturing by using metabolic processes and reducing agents found in bacteria, such as proteins, enzymes, and other reducing agents [46, 47]. Actinobacteria are aerobic, non-motile, and primarily filamentous gram-positive bacteria that produce secondary metabolites such as antibiotics. Due to their detoxifying ability, they are immune to even the most harmful heavy metals. Toxic metal ions that are soluble are either destroyed or precipitated intracellularly or extracellularly. Antibacterial, antifungal, anticancer, antioxidant, anti-bio-contamination, and catalytic nanoparticles can thus be made [48] Nanoparticles can be synthesized extracellularly or intracellularly by enzymes using easily cultured and fast-breeding eukaryotic yeasts and fungi, as well as simple biomass design. The size of the nanoparticles produced is influenced by the incubation conditions and the metallic ion solutions employed. Since some fungi are pathogenic to humans, they cannot be used to make nanoparticles [49]. Algae are eukaryotic aquatic photosites that use pigments, proteins, carbohydrates, lipids, nucleic acid, and secondary metabolites to break down metallic ions into nanoparticles. The algae extract, which is present in an aqueous medium at a specific temperature, is supplemented with metal solutions of the appropriate pH and concentration, resulting in the synthesis of nanoparticles with antibacterial characteristics without the production of hazardous by-products. Algae are additionally advantageous to this synthesis technique because of their widespread availability and applications [50]. Among all of these, plant-based nanomaterials have more facile and cost-effective methods of synthesis. Chemical synthesis of nanomaterials is thought to be easier due to the collection, and identification/maintenance of plants from field is required in green synthesis.

## 7. Salient Examples of Chemical- and Green-Synthesized Nanoparticles

### 7.1. Silver Nitrate

Sodium citrate, sodium borohydrate, and silver nitrate were employed as building ingredients in the wet-chemistry procedure. In the green synthesis process, leaf extract of *Nigella sativa* was utilized as a reducing and encapsulating agent to reduce silver nitrate. Furthermore, the effects of both produced AgNPs on bone-building stem cells in mice, as well as seed germination and vegetative growth in six different crops, were studied (wheat, bean, Lolium and common vetch, canola, and lettuce). The colorless reaction mixtures changed brown in both synthesis processes, and UV-visible spectra verified the existence of silver nanoparticles [51]. The harmful effects of the chemicals AgNPs and AgNO3 on *Brassica nigra* seed germination and seedling growth were explored. They discovered that while both chemical AgNPs and AgNO3 reduced lipase activity, seed germination, soluble and reducing sugar levels in germinating seeds, and seedling growth, chemical AgNPs had a greater impact. Chemical AgNPs inhibited *Arabidopsis thaliana* root elongation more effectively than AgNO3. Chemical AgNPs were found to be concentrated in leaves and disrupt chloroplast transcription of antioxidant enzymes and aquaporin genes at higher levels than AgNO3, implying that AgNPs are more hazardous than AgNO3 [52]. For agricultural use, green-generated AgNPs are preferable than chemically synthesized AgNPs. It is probable that AgNPs made with plant extracts are encased in a thin layer of capping organic material from the plant leaf broth and that this organic coating decreases their toxicity compared to those made with chemical procedures. Various methods for manufacture of AgNPs employing various materials as capping and reducing agents have been reported, and their antibacterial activities have been investigated. Green and chemically produced silver nanoparticles were compared, with the varying reducing and capping agents. Green nanoparticle manufacturing via bioreactions that result in the reduction of silver ions to particles could be a viable option without the use of extra reducing chemicals. Furthermore, the method's scale-up operations are simpler than chemical and physical synthesis approaches, making it more efficient. Green AgNPs were discovered to be spherical, isotropic, and stable.

Silver nitrate solution against two species of Hyalomma (*H. a. anatolicum* and *H. m. isaaci*) showed 12.25 and 12.17 mg/L of LC_50_, while LC_90_ stood 49.17 and 46.52 mg/L, respectively. Highest efficacy in terms of LC50 and LC90 was noted against *H. a. anatolicum* in their study [53]. In another study, LC_50_ of AgNPs were noted to be 28.96 against *Haemaphysalis bispinosa*, while these lethal concentrations were 31.02 mg/L against *Hippobosca maculata* [54]. They also showed strong antibacterial activity, and because chemically generated ones had oxidative activity, green-AgNPs could become a useful component than of chemical nanoparticles for medical applications. The DNA cleavage activity of green-AgNPs has also been verified, and this mechanism could be responsible for the antibacterial activities. Green nanoparticles, for example, had a narrow size range, a spherical shape, and high antioxidant, antibacterial, and DNA cleavage activities, compared to chemically synthesized nanoparticles, which had a smaller average size, a larger range of nanoparticle sizes, no antioxidant activity, and lower antibacterial and DNA cleavage activities. As a result, green produced silver nanoparticles have the potential to be used as a biological agent on microbes and bacteria in the environment [55, 56].

### 7.2. Iron Oxide Nanoparticles

The manufacture of iron oxide nanoparticles (FeONPs) is becoming more popular as a result of its magnetic properties and potential uses in a variety of contemporary technologies. FeONPs are vital in biomedical applications such as diagnostics, therapy, and medication administration, as well as bioremediation. Considering biological methods are environmentally beneficial and chemical methods are hazardous, the synthesis pathway of nanoparticles is a big concern. The FeONPs were synthesized using two different processes, chemical and green, and their characteristics and performance in applications were compared. FeONPs were made utilizing both a green synthesis approach and a chemical process using *Cardiospermum halicacabum* ethanolic extract (CHE). UV-vis spectroscopy, XRD, FTIR, particle size analyzer, vibrating-sample magnetometer (VSM), and high-resolution scanning electron microscope (HRSEM) analyses were used to characterise FeONPs. The FeONPs had a rhombohedral structure, with sizes of 20.9 and 42.3 nm, correspondingly, for green and chemical synthesis of FeONPs. Due to the sheer capping and stabilizing compounds included in the CHE, the green synthesis FeONPs exhibited better properties than chemical synthesis FeONPs. Additionally, cell survival and intracellular ROS scavenging experiments revealed that the green-synthesized FeONPs were biocompatible. In addition, as compared to chemically synthesized FeONPs, green synthesis FeONPs had greater bactericidal efficacy. The nanoparticles generated are also ecologically friendly, as demonstrated by the brine shrimp lethality assay. Thus, green-synthesized iron oxide nanoparticles can be a viable option that can be further studied for their prospective applications in many biomedical applications, particularly drug delivery and efficient nanopesticides applications [57]. Iron works by two ways depending upon its source of breakdown from the source. Breakdown of hemoglobin releases iron which is strong generator of reactive oxygen species (ROS). On the other hand, nonheme source, i.e., inorganic iron and ferric transferrin, released from host transferrin is ultimately producing ROS [56, 58].

#### 7.2.1. Nanopesticides from Arthropods

Arthropods are members of the phylum Arthropoda, which includes insects, arachnids, myriapods, and crustaceans. Arthropods have specific characteristics such as hinged limbs and an exoskeleton comprised of calcium carbonate and alpha chitin [59]. Naturally sourced are typically bioactive components, and their derivatives account for particularly half of the medications utilized therapeutically to treat a variety of disorders around the world. Many of these items are derived from plants, fungus, and microorganisms (N. [60]). Furthermore, it has been noted that, due to their global prevalence, arthropods have also played significant role in the implementation of abundant and cheap therapeutic medicines, particularly in economically challenged countries. From long period of time, honey is a biological product of the honeybee, has been acknowledged as a NutraSweet product of humans, and is also regarded as being one of the highly recognized nutritious foods. Additionally, because of their achievements to the advancement of nanotechnology, the boundaries of understanding about bees and producing honey have been enlarged. Several modifications for green manufacturing of various metallic nanoparticles (MeNPs) have been made to enhance their biodegradability, cost-effectiveness, safe practices, and inevitably less toxic effect when compared to physical and chemical methods. Furthermore, in recent years, nanoparticles has made inroads for animal species, notably insects and their by-products, to be employed as great options for the green synthesis of metallic NPs [61]. Considering the need for natural compounds that are safe, environmentally friendly, and cost-effective in the composite of metallic NPs, bees and their honey have been employed to facilitate the biogenesis of various metallic NPs such as gold (Au), silver (Ag), and oxides of cerium and copper (CeO and Cu_2_O) [61]. The characteristics of some of nanopesticides are given in Table 2.

#### 7.2.2. Bees and Bee Honey

**Figure 9:**
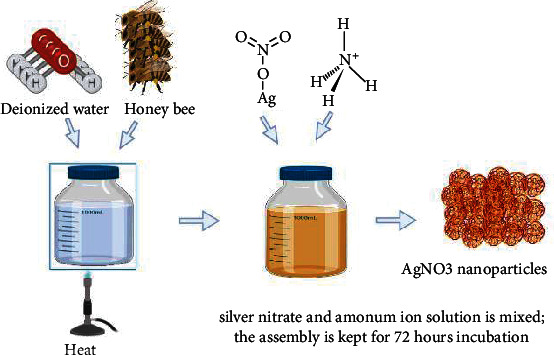
Production of silver nanoparticles with use of bee honey. Honeybee is mixed with deionized water and heated to make soluble solution. Silver nitrate and ammonium solution is added and kept for 72 hours, a yellow colour solution. Silver nitrate nanoparticles in dark brown form are obtained finally.

Silver is a harmless inorganic substance that has been shown to inhibit more than 650 different types of bacteria [62] and has the highest thermodynamic properties being a metal [63]. AgNPs have the highest level of commercialization among nanoparticles and have a lot of applications [64]. For example, of the several nanoparticles studied, AgNPs (Figure 9) are the most potential nanoparticles employed in nanomedicine due to their antibacterial activity against a variety of microorganisms [65]. N. Singh et al. [60] used bee honey to illustrate the production of AgNPs (Figure 9). Honey was heated in deionized water and then mixed to a solution of silver nitrate (AgNO3) and ammonium at a concentration of 1 mm. The transformation of the solution's colour from bright yellow to dark brown after 72 hours of response showed the creation of nanoparticles. When exposed to UV-vis spectroscopy, the biologically synthesized AgNPs were maximum absorbed at 420 nm. This peak revealed the exogenous decrease of silver ions. The Fourier-transform infrared (FTIR) spectrometry of the AgNP solution showed proteins as the biomolecules required for the biologically synthesized AgNPs' stability. Analysis approaches such as UV-vis spectroscopy, X-ray diffraction (XRD), and scanning electron microscopy were used to analyze the biosynthesized AgNPs (TEM). Maximum absorbance at 413 nm was detected, as well as monodispersed circular shaped nanoparticles with size of about 4 nm (as revealed by TEM). The biosynthesized nanoparticles were identified as nanocrystallites with a size of 6 nm by XRD. Although glucose present in honey has been implicated in the degradation method, proteins in honey have been reportedly liable for AgNP stability.

#### 7.2.3. Production through Cobwebs

**Figure 10:**
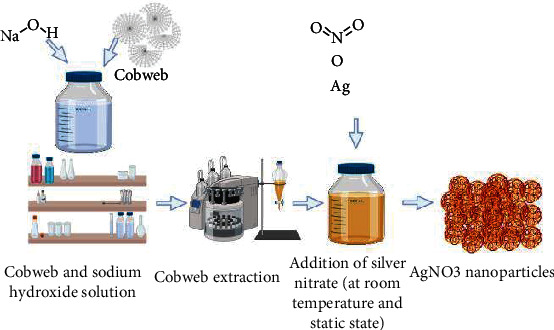
Production of silver nanoparticles with use of cobweb. Cobweb and sodium hydroxide are dissolved to make solution. Extracts of cobweb are made. Silver nitrate is added at room temperature and kept at static position. Silver nitrate nanoparticles are prepared in dark brown form.

The first study of the usage of spider cobweb as a unique biological matter for AgNP synthesis (Figure 10) to the extended constraints of knowledge on biotechnological processes of biosynthesized AgNPs [61]. The cobweb extract was treated with a 1 mm AgNO3 solution after being alkaline hydrolyzed with NaOH under suitable circumstances. The reaction (extract/AgNO3 solution) produced a dark brown colour as it progressed under stable conditions (at room temperature). Analytical techniques such as UV-visible spectroscopy, FTIR, EDX, TEM, and SAED (selected area electron diffraction) examination were used to evaluate the biosynthesized nanoparticles. The greatest absorbance of the AgNPs produced was at 436 nm. Arthropods clearly have powerful biomolecules that can be used to bioreduce the metal ions to their metallic states. The various biologically active compounds found in arthropods and their metabolites can be used to create new materials for the green manufacturing of nanoparticles [61].

## 8. Applications of Salient Metallic Nanopesticides

Ideally, green metal NPs, like Ag, Ti, Zn, Ni, and Cu, interceded by plant-based compounds, are less expensive and single-step reaction and do not need harmful substance compounds. Nanoformulations likewise shield the dynamic mixtures from ecological debasement and empower fast infiltration into ticks. Also, laying twise and controlled pesticide discharge advancements utilizing nanomaterials could increase pesticide-stacking, work on the dispersibility and dependability of dynamic fixings, and advance objective capacity. From the “one-wellbeing” viewpoint, such shrewd details could address novel eco-accommodating arrangements in the wake of uncovering their ecotoxicological profiles for the security of the human, creature, and nontarget living beings [23].

### 8.1. Zinc Oxide Nanopesticides

The mechanism of toxicity induced by ZnO NP may be attributed to the production of cell damage, leading to release of ROS (reactive oxygen species). Also, the release of Zn ions on contact with epidermis of live organism may lead to initiation of toxicity pathway [66]. A study employed the use of indigenous plants (neem and lemon grass) leaves for synthesis of ZnO NP to evaluate efficacy and toxicity within lower lethal concentrations. Also, the study is focused to determine *in vitro* effects of ZnO NPs against various life cycle stages in one of most important and under explored tick genera—*Hyalomma*, *Haemaphysalis*, and *Rhipicephalus*. Green nanoparticles of ZnO have been utilized for applications against a variety of parasites and disease vectors, viz., mosquitoes, ticks, lice, and flies ([18]; Ng *et al*., [67]). It is proposed that nanoparticles work for toxicity induction by either accelerating or decelerating some cellular mechanisms of host cells [68]. The unique physicochemical surface properties of nanomaterials make them more suitable for downstream functionalized applications. In addition to that ZnO NP are classified as generally recognized as safe (GRAS) by the United States Food and Drug Authority (FDA), making them one of safest nanoparticles for biomedical applications [69]. The safety profile of ZnO NP during *in vivo* trials was validated by running serum biochemical analysis of subject animals (Arafa *et al*., [70]). Similarly, in humans, the failure of ZnO NP to induce toxicity or by-pass cellular layers was recorded 5 days postexposure (Mohammed *et al*., [71]). Until recently, only N-doped TiO_2_ NPs became used to suppress pathogens related to agriculture. Furthermore, certain other nitrogen-doped nanoparticles with similar photochemical disinfection of pathogenic microbes, such as nitrogen-doped ZnO and carbon dots, are not commonly used. Because of their outstanding antibacterial and antifungal activities via photocatalysis, nitrogen-doped nanoparticles have the capability to be used in pest control. This form of nitrogen-doped ZnO nanoparticles, like N-doped TiO_2_ NPs, often exhibits better photocatalytic capabilities than the pure ZnO ([72, 73].

### 8.2. Gold Nanoparticles (AuNPs)

Metal NPs, especially AuNPs, show exceptional biocompatibility and low toxicity. Furthermore, because it is lesser reactive than silver, it could be employed for long-term purposes. Philip [74] revealed that honey can be used in the production of AuNPs. Raw honey become diluted in deionized water before being treated with a solution of gold chloride (HAuCl4). The AuNPs generated after the reduction of metal ions of AuCl4 have been light purple in colour, with the highest absorbence measured at 541 nm. As the amount of honey raised, the magnitude of the nanoparticles diminished. According to TEM, the nanoparticles created have anisotropic shapes of sphere, rod, and triangle. XRD (which validated the crystal-clear nature of the biologically synthesized Au nanoparticles with an averaged magnitude of 15 nm) and FTIR were also used, identifying protein as the wrapping and stabilizing biomolecule. Because of their photoluminescence properties, the nanoparticles have also been proposed for medicinal usage. Because of the bioconjugation with honey, the AuNPs emitted photoluminescence at 447 nm.

### 8.3. Silver Nanoparticles against Ticks

The silver nanoparticles (Figure 6) are required as against bacterial specialists specifically with antimicrobial species that are resistive. Silver- (Ag-) covered therapeutic gadgets, nanogels, Ag-based dressings, and so forth, as contrasted and Au, Ti, Zn, and Mg, are viewed as the most proficient against microbial specialists, yet when Ag NPs are utilized as a sanitizer, these lead to worries with respect to argyrosis, harmfulness in mammalian cells, and argyria. This outlines the significance of feature of biogenic silver nanoparticles and AgCL to comprehend the activity on ticks or microbes communicated by them. Antitick activity of silver chloride nanomaterials has been noticed against Rhipicephalus (Boophilus) microplus, Hyalomma anatolicum, Hyalomma isaaci, and Haemaphysalis bispinosa [75]. A better option for treatment of these illnesses is the utilization of nanobiotechnology as a clever technique around here. One huge nanostructure is the silver nanoparticles (Ag) or moreover nanoparticles of the silver chloride (AgCl). The silver nanoparticles biogenically consolidated are by and large considered by numerous assessment bundles in the world and AgO nanoparticles were extremely useful against *Aedes aegypt*i. The biogenic silver nanoparticle ramifications for ticks were thought about against ticks' hatchlings and adult ticks [76]. Silver and silver chloride NPs are efficient hostile to ticks; that rely upon the creation of nanoparticles and from their shapes. Because of this element, AgCl in its circular morphology showed less action than circular AgO. Another factor to be considered while assessing the impacts of nanomaterials is the efficiency of proteins forming biogenic nanomaterials. This might be due to specificity of protein interaction that may influence the applications of the nanomaterials [77].

### 8.4. Nanopesticides Based on Nitrogen-Doped TiO_2_ NPs

A new agri-tech trend had recently begun to increase agricultural capacity to achieve the world's growing food demand. Engineered nanomaterials have the capability to have a positive impact on the environment while also improving agricultural efficiency, resilience, and sustainability. Nitrogen-doped nanoparticles (N-doped NPs) are a prominent figure in the emergence and progression of nanopesticides for efficient and sustainable agriculture, providing efficiency improvements, novel insects control notions, and lower acaricide resistance, all of that are major constraint of agrochemicals. The key benefits of N-doped NPs are their multifunctionality, which allows for improved adherence on leaves or insect bodies, as well as multiple various modes of action to kill insects, including biochemical, catalytic, and physics which are projected to significantly lessen insects. Beside insects, these nanomaterials can inhibit the activity of phytopathogenic bacteria and fungi via a variety of processes and are thus useful for a wide range of plant protection. Because of their outstanding photocatalytic property, which generates diverse reactive oxygen species, TiO_2_ NPs became widely employed in reducing the growth of weed, bacteria, fungi, and other plant diseases (ROS). This characteristic has also been greatly enhanced by doping with nitrogen to destroy some bacteria and fungus. For instance, nitrogen-doped oxide of metal nanocatalysts like TiO_2_ improved the photodegradation performance of *E. coli* bacteria significantly [78]. Particularly, a test was carried out in the dark to study the associations of titanium oxide and nitrogen-doped TiO_2_ nanoparticles including *E. coli* bacteria. Therefore, photoelectrocatalytic nanoparticles have no noticeable effect on bacterial reduction. Alternatively, N-doped TiO_2_ exhibits a significant rise in neutralizing *E. coli* bacteria after 120 minutes of light illumination. Other investigation is being conducted to examine the effectiveness of various nitrogen supplies in suppressing E. coli germs. The results show that N-doped TiO_2_ nanoparticles utilizing ethylenediamine as a precursor inhibited the growth of E. coli the best within 90 minutes. In the meantime, N-doped TiO_2_ NPs produced from ethanolamine have shown a substantial betterment in *E. coli* inhibition. It is understood that this is due to the Ti-N bonds that reside in N-doped TiO_2_ NPs. Furthermore, it is thought that the Ti-N bond facilitates electron interaction between the titanium of TiO_2_ NPs and nitrogen in the doping, resulting in a shift in electron structure near the valence band edge. As a result, TiO_2_'s energy difference has shrunk [79]. Another piece of evidence, in particular, has been cited to describe the photocatalytic inactivation process of E. coli employing N-doped TiO_2_ [80].

## 9. Applications of Salient Nonmetallic Nanopesticides

### 9.1. CDs as Powerful Nanopesticides

**Figure 11:**
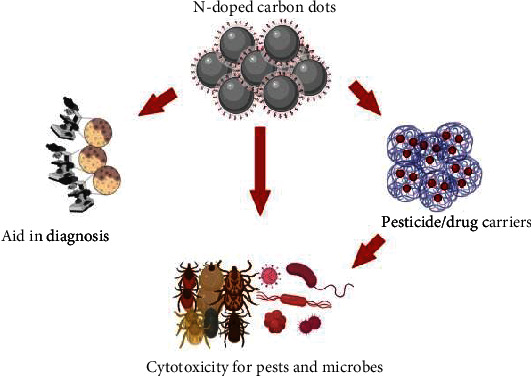
Applications of N-doped CDs (nitrogen-doped carbon dots). N-doped CDs are used in diagnosis of diseases; work as carriers for drugs that kill microbes and pests; and have direct effect on pests and microbes.

Carbon quantum dots (CQDs), also known as carbon dots (CDs) or carbon nanodots (CNDs) (Figure 11), are one of the founding additions of the carbon-based nanomaterials family ([81, 82]. CDs are distinct and quasispherical carbon nanoparticles with nanosized particles ranging from 1 to 10 nm ([83, 84]. Their distinguishing characteristics are the combination of distinctive fluorescence qualities of quantum dots and good electrical resources of nanoscale materials. Furthermore, CDs exceed other conventional substances regarding cost, nonpoisonous, luminous nature, and potential for forming -/h+ pairs when exposed to UV radiation [85]. It has also been established that CDs can block the activation of *E. coli* bacteria in the presence of oxygen ([82, 86]. They are hence hoped to be best used against livestock pests because of their activity against wider range of pathogens.

## 10. Pros of Nanopesticides

Nanopesticides could have the advantage of having little effect on nontargeted organisms and being environment friendly. They can be made using a variety of processes, including chemical, biological, and green synthesis.

The following are the most prevalent advantages of nanoparticle-based pesticide formulations:

- Improved formulation consistency
- Improved water solubility of active substances that are not water soluble
- In contrast with the commonly used insecticides, hazardous organic solvents are eliminated
- Ability to release active substances in a sustained manner
- Enhanced stability to avoid early deterioration
- Because of the reduced particle size, there is increased mobility and insecticidal activity
- Greater surface area, helping them offer enhanced action and last longer [26]

Nanopesticides' enhanced bioavailability and solubility may have an impact on their environmental fates, along with their chronic toxicity and dynamic characteristics when consumed by organisms. Stronger bioavailability, for example, could have a stronger effect on nontarget organisms. In the same way, reduced nanopesticide decomposition may cause unpredictable harm to nontarget organisms [87]. There is also indication that present nanopesticides and traditional pesticides behave differently in the environment, necessitating a thorough understanding of nanopesticide destiny in order to ensure compliance with regulatory norms and regulations [88]. Since commercial preparations may differ in terms of unidentified constituents, comparative toxicity research of their commercial and technical-level preparations seem to be required for a more precise and suitable risk evaluation [89]. As a result, a thorough toxicological evaluation of the potential dangers connected via the use of nanopesticides is required [90].

## 11. Barrier to Use Nanopesticides

The potential negative effects of nanoparticles on the environment are yet unknown, and determining the dispersion and behavior of nanopesticides both during and after application towards the environment is crucial for understanding their potential influence on ecosystems ([91, 92]. Comparison of nanoformulations with the pure active ingredient and standard formulations is required to understand how nanoparticles influence pesticide activity.

### 11.1. Complex Test Methodologies Required

Nanopesticides are anticipated to act differently than traditional insecticides, and a few of the typical ERA measures may be inapplicable to nanopesticides. As a result, additional test methodologies and metrics may be required to evaluate the environmental hazard of nanopesticides. Although it may be feasible in the future to expand rigorous testing procedures and modelling strategies that precisely calculate the sensitivity and impacts of a nanopesticide in a specific circumstance, it is also necessary to remember that existing methodologies for traditional evaluation of pesticide danger that use numerous presumptions in order to resolve ambiguities are far from flawless. To go forward in a realistic and executable manner, a logical approach that compensates for the basic distinctions amongst nanopesticides and conventional pesticides as well as more evidence for its promise at herd scale may be required [93]. It has already been known that nanobased pesticides have different transport, bioaccumulation, and biodegradability than conventional pesticides. There is a deficit of evidence on the impacts of nanobased pesticides on crop health, soil biodiversity, nontarget species, and human health [32, 94].

### 11.2. Future of Nanopesticides

Even though nanotechnology has numerous potential benefits against ticks, there is no nanotechnology-based product available in the market till date. One aspect contributing to this low level of industrialization is that the vast number of ongoing research is still being conducted at universities and research institutes, or by tiny enterprises (spin offs and start-ups) to establish the cost-effectiveness of nanomaterials at herd level. At around the same time, huge corporations own a vast number of patents, with the number increasing year after year. New nanobased goods do not reach the market because huge corporations accumulate trademarks and wait for prospects for future utilization following the progress of attractive commercial items [95].

- Consumer awareness and acceptability
- Scalability
- Funding agencies are required to buy these ideas
- Ease, frequency, and dose of application may be considered
- Studies on nontarget effects on host animals and other organisms should be encouraged

### 11.3. Economic Issues and High Cost Related to the Use of Nanopesticides

In contrast to the limitations outlined above, it really should be noted that economic concerns continue to be a key impediment to the development of nanopesticides. The initial expenses of creating nanopesticides are significant, with positive financial returns conceivable only if huge quantities of these chemicals are utilized, which is far from being practical yet. Furthermore, a lack of regulation is a key hindrance to the growth of nanotechnology in livestock. Another barrier to the development of nanobased insecticides is the high expense of establishing a novel active ingredient [96].

## 12. One Health Concerns of Nanopesticides

Nanoparticles have become equally important for bacteria [97] as well as ticks. As the application of nanopesticides became more prevalent, problems about how to evaluate the ecological hazard of these resources have arisen. The present methods for assessing pesticide ecological danger are reread, and subject of whether these tactics are adequate for practice with nanopesticides is discussed a lot. The susceptibility of nanoscale-based compositions and their environmental consequences are major concerns that must be addressed. The number of nanoformulations in soil, surface water, and groundwater, as well as their effects on nontarget organisms, is not well known. The fate of nanoformulations may be influenced by a variety of chemical aspects such as pH, ionic strength, and the concentration of dissolved molecules in the environment. Off-site movement of pesticides in the dissolved form reduces pesticide runoff to surface streams, while corrosion of the external earth can also donate to aquatic pollution.

The toxicity of a variety of pesticides used in nanoscale compositions has been determined by specialists, and the possible adverse effect as well as the possible impacts of nanodimensions must be considered during nanoscale formulations. More research is needed to confirm the toxicity of nanoscale formulations and the components that contribute to nanoparticle toxicity, such as size, charge, shape, and chemistry. The lifetime of nanopesticides in the environment is the most important criterion for assessing the risks associated with their use. Nontarget organisms are exposed when liberation or release is halted for an extended time. One of potentially adverse effects of the nanocarriers is that they promote the transfer of some of the immobilized operational components. As a result, organisms might have more exposure to them. Certain nanoformulations have been shown to improve absorption by target species. It must be verified that no nontarget organisms are harmed.

Insecticides are examined like other toxins are evaluated. Since the Trojan horse action causes a nanopesticide to collaborate with other pollutants, hazard analysis of ecological interaction is often not estimated. As a result of the interaction of the chemical with ENP, the substance is delivered to a tissue of the animal or from an organism, enhancing the interior exposure to pollutants and possibly preventing accumulation. The moment has arrived to integrate these interconnections into authoritative risk assessment systems [98].

## 13. To Do List

- Engineered nanoparticles (ENPs) are employed in a lot of industries, counting protection, dynamism and packing, cultivation, or ecological cleanup, or have the power to be effective in the future. With the expansion of a spectrum of herbal safety harvests known as “nanopesticides,” one part where the usage of ENPs is getting momentum is the insecticide industry
- There are few instruments and techniques for evaluating the characteristics of complex nanopesticide formulations. Supervisory supplies for information on hazzard calculation cannot be achieved without appropriate analytical tools. Current ecotoxicity analyses are limited in their value due to the inability to describe ENPs in complex environmental compartments, despite the fact that this field is rapidly evolving. Discovery and measurement of nanoparticles and metallic oxides in multifaceted backgrounds at practically realistic amounts remain hard, despite heightened awareness to artificial ENPs
- Nanotechnology of pesticides may also aid good agricultural practices in the future over “clever ground methods”; for instance, wireless devices might be related to a personal processer via satellite to notice and find pathogen outbreaks in crops and trigger pesticide spraying as wanted. These technologies take the ability to eliminate the need to put on an insecticide to the whole yield, thus conserving air quality and reducing pesticide application volumes and focusing pesticide application
- PECs in earth, ground water, and squeezed water should be determined as ERA objectives under existing rules. It is important to note that flow-based models pour pesticides to soil outline where leakage or overflow to the environment is quite possible despite of the accurate pore sizes of the models.
- In many parts of the globe, ecological evaluations of pesticides are needed before a product can be put up for sale. The approximation of a forecast ecological attentiveness, which is the anticipated intensity of lively agent in important ecological sections such as surface water, ground water, and earth, will be required at each tier.

## 14. Conclusion

Ticks cause enormous economic losses to cattle and have several detrimental impacts on the animals they infect. Nanotechnology has solved several of agriculture related issues important of which is pest management. The nanopesticides in this regard may be used as an excellent candidate to control pests. A diverse range of methods of production, mode of actions, and routes and forms of applications of nanopesticides on target species have made these a worth appreciating novel approaches where drug resistance is at rise. One health concern associated with the application of nanopesticides beyond bench side needs to be prioritized in research, wherein the safety evaluation of NPs is crucial. The off-target effects, safe environmental application, and degradation of NPs need to be carefully calculated to convince the farmers/end consumers and environment regulatory authorities for a paradigm shift towards NPs at farm scale.

## Figures and Tables

**Table 1:** Comparative analysis of chemical synthesis and green synthesis of nanopesticides.

Green synthesis-based nanoparticles	Chemical-based nanoparticles
Plants, seaweeds, seagrasses, and microorganisms are examples of biological sources that are both cost-effective and have fewer negative effects.	Chemical-based NP manufacturing is both costly and detrimental to the environment.
Plant-based nanoparticles do not need a lot of pressure, energy, or heat to work and remove harmful chemicals.	At room temperature, most chemical-mediated nanoparticle production procedures are carried out.
The key benefit of green synthesis nanoparticles is that they are environmentally beneficial because they use waste materials as a raw supplement in the synthesis process. This technique's raw resources are all renewable.	Chemical-mediated synthesis of nanoparticles has the fundamental advantage of allowing the manufacture of particles with defined size, dimension, composition, and structure, which can be applied in a variety of study areas.
Nanoparticle synthesis from terrestrial plants is a relatively simple process since it eliminates the need to change the liquid medium. To make silver NPs, aqueous plant extracts of Matricaria recutita were employed.	Silver NPs were synthesized at 55–60°C using two stabilizing agents, polyvinylpyrrolidone (PVP), and gelatin, using various sugars such as glucose, fructose, lactose, and sucrose.
These nanoparticles range in size from a few nanometers to around 100 nanometers.	These nanoparticles range in size from 25 to 450 nanometers.
Low yield is the fundamental drawback of green nanoparticle synthesis.	Chemical-based nanoparticle can induce inhalation issues and a variety of deadly diseases due to their minuscule size.

Deepak et al. [36].

**Table 2:** Characteristics of salient nanopesticides prepared from arthropods.

Bioactive material	Metal	Metallic nanoparticles	Maximum absorbance wavelength (nm)	Shapes of the nanoparticles
Bee honey	Silver (Ag)	AgNPs	UV-vis spectroscopy and FTIR show max absorbance at 420 nm	Revealed by TEM circular shape 4 nm, XRD nanocrystallity 6 nm
Bee honey	Gold (Au)	AuNPs	UV-vis spectroscopy max absorbance 541 nm	TEM shows anisotropic shape of rod, sphere, and triangle XRD shows crystalline shape with 15 nm
Cob web	Silver (Ag)	AgNps	UV-vis spectroscopy and SAED show max absorbance at 436 nm	3-50 nm, spherical

FTIR: Fourier-transform infrared; TEM: transmission electron microscopy; XRD: X-ray crystallography; SAED: selected area electron diffraction.

## Data Availability

All data is included in the article.
